# Supplementary material for: Relationship between glass transition temperature, and desiccation and heat tolerance in Salmonella enterica
Source: PLoS One. 2020 May 29;15(5):e0233638. doi: 10.1371/journal.pone.0233638 (PMC7259547; doi:10.1371/journal.pone.0233638)
Supplement: Attachment — Submitted filename: Responses to the reviewers_PONE-D-20-08834.docx [file pone.0233638.s001.docx]

May 1, 2020

**PONE-D-20-08834**

**Title: Relationship between glass transition temperature and desiccation/heat tolerance in *Salmonella enterica***

Dear Dr. Sant'Ana,

Academic Editor, PLOS ONE,

Thank you for your email on April 22, 2020 regarding our manuscript PONE-D-20-08834. We greatly appreciate the reviewers’ comments and suggestions that have helped us improve our manuscript. We have revised the manuscript corresponding to the reviewers’ comments. Our point-by-point responses to the reviewers’ comments are appeared below.

**Reviewer #1**

Summary: In this study, Lee et al investigate the relationship between glass transition temperature (Tg) and desiccation and heat resistance in Salmonella. The main claim of the paper is that the heat and desiccation resistance of Salmonella can be attributed to the inherent Tg of the strains. This is an interesting hypothesis and has relevance to the field however, there are a number of major issues (see list below) that require attention before this manuscript can be published.

***1. Conclusions not being supported by data
- The authors assert that the observed differences in heat resistance are caused by differences in Tg however the data presented do not support this claim. The relationship between Tg and aw and aw and heat resistance is shown, but the relationship between Tg and heat resistance is not. There could be many other factors unrelated to Tg that can explain the heat resistance results.***

**RESPONSE**

⇒ Thank you for your critical comment. In fact, there was no direct relationship between Tg and heat resistance in the original manuscript. Based on the relationship between Tg and aw (Fig. 3 in the original manuscript), and the relationship between aw and heat resistance (Fig. 4 in the original manuscript), we considered that the relationship between Tg and heat resistance would be illustrated indirectly. However, as the Reviewer#1 concerns, it would be unclear the relationship between Tg and heat resistance. Accordingly, we have added one more figure that illustrates directly the relationship between Tg and heat resistance (log reduction) as a new Figure 4 in the revised manuscript as shown below. The result shows the trend that bacterial inactivation effect increases with decrease in Tg, although there is variation among serovars.

Fig. 4. Relationship between glass transition temperature (*T*_g_) and inactivation effect (log reductions) of *Salmonella* Typhimurium (○, solid line), *S*. Chester (△, dashed line), *S*. Oranienburg (□, dotted line), *S*. Stanley (◇, fine dotted line), and *S*. Enteritidis (▽, dot dashed line).

**“To determine the relationship between *T*_g_ and heat resistance (log reduction) of *S. enterica* serovars, we illustrate both the relationship as shown in Fig. 4. There seems to be *T*_g_ dependency on heat resistance, which means bacterial inactivation effect increases with decrease in *T*_g_ for the tested *S. enterica* serovars except for *S*. Stanly. The correlation coefficient of *S.* Typhimurium, *S*. Chester, *S*. Oranienburg, *S*. Stanley, and *S*. Enteritidis is -0.54, -0.75, -0.80, 0.01, and -0.99, respectively. The result would also support that the *T*_g_ plays an important role in heat resistance of *S*. *enterica* cells in dry conditions.”** (Please see Lines 212-218 in the revised manuscript.)

***- Contrary to line 186, Salmonella ser. Stanley has an Tg less than 60 in three of the four aw conditions. It also has the highest heat tolerance. This is contradicts the hypothesis and the main assertion of the paper (lines 234-235)– please discuss.***

**RESPONSE**

⇒ Thank the Reviewer#1 for the critical comments. As the Reviewer #1 indicated, the data presented in the original manuscript would lead to some confusions, because of the incorrect descriptions on the figure caption of the Figure 3 due to our careless mistake during revising the figure. Accordingly, we corrected the figure captions, however, we re-arranged the results based on the Reviewer #1’s another comments below regarding transforming Fig. 3 into a Table. Please see the Response to the comment 2.2 in below.

According to the correction, *S.* Stanley has higher glass transition temperature at a_w_ 0.87, unlike other *Salmonella* strains. As shown in Fig. 4 in the original manuscript (the figure will refer to Fig. 3 in the revised manuscript), when heat treatment of 60 ºC was applied, the *Salmonella* strains other than *S.* Stanley showed a higher log reduction at a_w_ 0.87 than those of other a_w_ levels. The difference in the bacterial inactivation effect between aw levels would be related to the Tg. In contrast, *S.* Stanley did not change log reduction of bacteria depending on increasing water activity. This is considered that *S.* Stanley has a high glass transition temperature even at high water activity, and as a result, it is high possibility to be in a glass state at a temperature of 60 ºC in a_w_ 0.87, and thus has high heat resistance.

***2. Technical issues
2.1- The aw of the stored cells should be confirmed prior to measurement of the Tg***

**RESPONSE**

⇒ The water activity and temperature in the air-tight container were always checked with a temperature recorder, and the water activity of the bacteria was confirmed with a water activity meter. Therefore, we have added the following text.

***“* The water activity and temperature in the air-tight container were continuously checked using thermo recorder (TR-72wf, T and D, Nagano, Japan). And the water activity of the bacteria was confirmed by a water activity meter (Aqualab 4TE, Decagon Devices, Washington, USA).”** (Please see Lines 117-120 in the revised manuscript.)

***2.2 Figure 3 could be better presented as a table so all of the values can be compared. Also it would be helpful if there was an analysis of significant differences in Tg at different aw***

**RESPONSE**

⇒ Thank you for your suggestion. We have changed Fig. 3 into Table 1 as fallows to add the information regarding the significant difference between a_w_ levels in each *Salmonella* serotype as well as the difference between *Salmonella* serotypes in each a_w_ as shown in the original figure.

Please see the Table 1 as shown in below in the revised manuscript and Lines 178, and 185 – 191.

Table 1. Relationship between water activity (a_w_) and observed glass transition temperature (*T_g_*) of *Salmonella enterica* serovar Typhimurium, *S*. Chester, *S*. Oranienburg, *S*. Stanley, and *S*. Enteritidis.

| *S. enterica*  serovars |  | Glass transition temperature (Tg, °C) | | | | | | | | | | | | | | |
| --- | --- | --- | --- | --- | --- | --- | --- | --- | --- | --- | --- | --- | --- | --- | --- | --- |
|  |  | 0.43 aw | | |  | 0.57 aw | | |  | 0.75 aw | | |  | 0.87 aw | | |
| *S*. Typhimurium |  | 81.63 | ± | 11.28^aA#^ |  | 70.39 | ± | 3.26^abA^ |  | 58.46 | ± | 2.23^abB^ |  | 35.16 | ± | 2.92^bC^ |
| *S*. Chester |  | 83.29 | ± | 2.04^aA^ |  | 75.62 | ± | 2.64^aA^ |  | 62.17 | ± | 1.37^bB^ |  | 43.50 | ± | 2.35^cC^ |
| *S*. Oranienburg |  | 81.26 | ± | 6.46^aA^ |  | 73.14 | ± | 1.95^abB^ |  | 63.39 | ± | 4.98^bC^ |  | 43.00 | ± | 1.33^cD^ |
| *S*. Stanley |  | 80.07 | ± | 2.96^aA^ |  | 65.94 | ± | 3.64^abB^ |  | 62.22 | ± | 3.31^abB^ |  | 57.46 | ± | 8.06^bB^ |
| *S*. Enteritidis |  | 77.10 | ± | 1.78^aA^ |  | 51.25 | ± | 2.89^bB^ |  | 45.37 | ± | 2.83^cBC^ |  | 40.69 | ± | 2.76^cC^ |

^#^Different lowercase letters in each a_w_ level represent statistically significant differences (P < 0.05) among the five *S*. *enterica* serovars. Likewise, the different uppercase letters in each *S*. *enterica* serovar represent statistical differences (P < 0.05) among four different a_w_ levels.

***2.3 Figure 4: significant differences of the heat resistance at different aw for each strain should be determined to allow for easier comparison***

**RESPONSE**

⇒ We appreciate the Reviewer #1’s constructive suggestion. We have added the information regarding the significant difference between a_w_ levels in each *Salmonella* serotype as different uppercase letters. Please see the revised Figure 4 referred as Figure 3 in the revised manuscript and the caption of the Figure 3 in Lines 220 – 227 in the revised manuscript as follows:

**“Fig. 3. Comparison of thermal inactivation effect (log reductions) of *Salmonella enterica* Typhimurium, *S*. Chester, *S*. Oranienburg, *S*. Stanley, and *S*. Enteritidis heated at 60 ºC for 10 min under 0.43 (a), 0.75 (b), and 0.87 (c) a_w_. Initial cell numbers (*N*_0_) right before heat treatment of dried bacterial cells are ranged 5 – 7 log CFU/mL depending on serovar and a_w_. Error bars represent standard error of the mean (n = 3). Different lowercase letters among the five *S*. *enterica* serovars at the same a_w_ level represent statistically significant differences (P < 0.05). Likewise, different uppercase letters among different aw levels for each *S*. *enterica* serovar represent statistically significant differences (P < 0.05). ”**

***2.4 It is not clear when the initial cell numbers for the heat tolerance assay were determined. From the way the paper reads, it seems as if the cells were at 10E7 before the freeze-drying/storage procedure. If this is the case, the cell numbers/viability need to be confirmed prior to the heat tolerance assay or else the results obtained could just be a reflection of cell viability after freeze drying***

**RESPONSE**

⇒ Thank you for the critical indication. As the Reviewer #1 mentioned, the description on the initial cell number in the original manuscript was unclear on the timing of determination of the initial cell numbers. The initial cell numbers we mentioned here were determined for the dried and a_w_-adjusted bacterial cells just before heat treatment. Depending on the *S. enterica* serovars, the viable cell numbers showed difference after adjustment of a_w_. That is why we evaluated the heat tolerance as log-reduction to minimize the difference of the initial viable cell numbers.

To clarify the meaning of initial cell numbers for the heat treatment experiment, we have modified the explanation of the method section.

“**In the same manner, dried bacterial cells (ca. 50 mg) were taken and determined the viable cell number as an initial condition.**”

Please see Lines 143 – 144 in the revised manuscript.

***2.5 A control of Salmonella at typical (> 0.99) aw would be helpful to have as a comparator for the Tg and heat tolerance results at low aw***

**RESPONSE**

⇒ In fact, glass transition phenomenon is observed only lower a_w_ conditions such as < 0.93. We could not determine the Tg for the tested Salmonella > 0.93 aw. Therefore, we focus on low water activity to explain mechanism of these strain that tolerate in low water activity condition.

Preliminary, some *S. enterica* serovars were examined at the same heat treatment in the a_w_ 0.99. We confirmed apparently higher inactivation effect of 6-7 log cycle reductions in a_w_ 0.99 than those of lower aw levels on the same heat treatment. Thus, we added the descriptions regarding inactivation effect at typical aw level as follows:

“**We preliminary examined thermal inactivation effect on some *S. enterica* serovars, and we confirmed apparently higher inactivation effect of 6-7 log cycle reductions in a_w_ 0.99 than those of lower a_w_ levels on the same heat treatment (data not shown).**”

Please see Lines 249-252 in the revised manuscript.

***2.6 A rationale for why the strains/serovars were chosen for this study is not given***

**RESPONSE**

⇒ Thank you for your suggestion. We selected these strains of *Salmonella* known to exist in dry foods and related to outbreaks. We have added it accordingly as follows:

**“Especially, various *Salmonella* serotypes were found in dry foods associated with outbreaks [1-12].”** (Please see Lines 42-43)

**“Finally, we aimed to resolve the relationship between the state change of several *Salmonella* serotypes that is known to be present in low water activity foods due to glass transition and the changes in thermal resistance in a desiccation environment.”** (Please see Lines 87-90)

***3. Other issues
3.1 Background: As someone who does not specialize in rheological measurements, I found that the information provided in the Introduction and Methods was insufficient and did not allow for the appropriate review of the methods used to determine Tg.***

**RESPONSE**

⇒ We added more detail descriptions as follows:

“Previous studies used by TRA investigated the effect of water content on the *T*_g_ of cookies (Kawai et al., 2014; Sogabe et al., 2018), hazelnuts (Ebara et al., 2018), and deep-fried food (Jothi et al., 2018). To conduct the measurements, a sample is compressed at a temperature below *T*_g_, and heated above *T*_g_ with compression. Then, the *T*_g_ of the sample can be determined as a force drop induced by the glass transition. This is a useful method to apply to amorphous powders. ”

Please see Lines 81-85 in the revised manuscript.

***3.2 There was little description of these measurements being done in a microbiological context and their applicability to bacterial cells. A discussion of the physiological state of vitrified cells would be helpful***

**RESPONSE**

⇒ To the best of our knowledge, the present study would be a first investigation based on the hypothesis that bacterial cells vitrify depending on environmental a_w_ level. Thus, there is few related literature and we could not discuss the physiological state of the cell beyond our speculation. Therefore, as only the related information, we discussed on tardigrades and sleeping chironomids that utilize cryptobiosis, are also resistant to high temperature, high pressure, as well as dry environments (Lines 262-264 in the revised manuscript). We could not discuss in detail, however, we do believe that the present study would be a trigger of further investigation of the vitrification of bacterial cells.

***3.3 are these cells considered to be analogous to VBNC (viable but not culturable cells)?***

**RESPONSE**

⇒ It is still unclear the relationship between glass transition and VBNC. However, we suppose that glass transition might be a part of VBNC state. When it becomes glassy state, the activity of the cells decreases, so it has something in common with the VBNC bacteria. However, it is necessary to study further because other factors besides water activity must be considered in order to judge these bacteria as VBNC bacteria.

***3.4 Line 211, Room temperature is generally 20 – 22 degrees C and not 30. This has implications concerning the assertion that Salmonella is in a glass state at room temperature***

**RESPONSE**

⇒ As a result of measuring *T*_g_ in this study, the *T*_g_ of *Salmonella* serovars showed more than 30 ºC when the water activity was ≤ 0.87. We consider that *Salmonella* would be glassy state in the room temperature (20-22 ºC) because room temperature was lower than *T*_g_ (>30 ºC). Thus, we have modified the sentence as follows:

**“These cells would be in a glass state under room temperature conditions, because room temperature (normally 20 -22 ºC ) is lower than those of *T*_g_ (30 ºC). ”** (Please see Lines 241 – 242 in the revised manuscript.)

Thank you again for the Reviewer #1’s helpful comments and suggestions.

**Reviewer #2**

***Reviewer #2: General comment:
The manuscript presents a study investigating the relationship or effect of glass transition temperature (Tg) under different water activity (aw) conditions using alternative thermal rheological analysis (TRA). The central aim was to test the survival of Salmonella strain in various conditions. This was based on a hypothesis that bacterial cells are vitrified in low-aw environments based on the physicochemical properties of solid particles.***

***Specific comment:
1. Topic should change to: Relationship between glass transition temperature, desiccation and heat tolerance in Salmonella enterica***

**RESPONSE**

⇒ We have replaced the title accordingly as follows:

**“Relationship between glass transition temperature, and desiccation and heat tolerance in *Salmonella* *enterica*”** (Please see Lines 1-2)

***2. Abstract did not present specific aspects of the result found. it only mention the result without details. For instance, the abstract must contain a summary of the result (temperature rising operation at aw 0.43, 0.57, 0.75, and 0.87). and the result for Tg (For example, for S. enterica Typhimurium Tg was 73.7 ºC, 58.6 ºC, 48.4 ºC, and 23.2 166 ºC at 0.43, 0.57, 0.75, and 0.87 aw, respectively).***

**RESPONSE**

⇒ Thank you for your suggestion. We have added next sentence as follows:

**“For example, while the *T_g_* of five *S. enterica* serovars was determined as 35.16ºC to 57.46ºC at 0.87 a_w_, the *T_g_* of all the five serovars increased to 77.10ºC to 83.30ºC at 0.43 a_w_. ”** (Please see Lines 28-30 in the revised manuscript.)

***Discussion:
3. Line 220-221: The current study observed a variation in Tg among bacterial species. There is need to offer a scientific explanation on why various species of bacteria reacted differently to Tg. Little explanation was offered in line 236, this is sufficient and convincing that all Salmonella strains have been shown to survive in specific conditions.***

**RESPONSE**

⇒ Thank you for your critical comments. In fact, we do not confirm any exact scientific reason/evidence for the difference among bacterial species/serovars. However, there might be two possible reasons. One of them would be due to properties that are genetically unique to the bacterial species/serovars. Another one would be due to properties that are acquired during survival in various harsh environments. In any case, the reason/evidence for the difference in Tg among species/serovars should be investigated in the future study.

“**The difference in *T*_g_ among bacterial species/serovars would attribute to innate (genetically) or acquired characteristics of each bacterial species/serovars. In particular, acquired characteristics might be due to habituation to various harsh conditions during survival process. To elucidate the exact reason for the difference in *T*_g_ among bacterial species/serovars, further genetical and/or bacteriological investigation will be needed in the future.** “

Please see Lines 274 – 277 and 284 – 286 in the revised manuscript.

***4. Line 223-224: In fact, it has been reported that the thermal inactivation effect of low-aw food also [34-37].
Should be corrected to
It has been reported that the thermal inactivation effect of low-aw food [34-37].***

**RESPONSE**

⇒ We have corrected accordingly as follows:

**“It has been reported that the thermal inactivation effect of low-a_w_ food [34-37]. ”** (Please see Lines 257 – 258 in the revised manuscript.

Thank you again for the Reviewer #2’s helpful comments and suggestions.
